# Effect of Hearing Protection Use on Pianists’ Performance and Experience: Comparing Foam and Musician Earplugs

**DOI:** 10.3389/fpsyg.2022.886861

**Published:** 2022-07-12

**Authors:** Elie Boissinot, Sarah Bogdanovitch, Annelies Bocksteal, Catherine Guastavino

**Affiliations:** ^1^Schulich School of Music, McGill University, Montréal, QC, Canada; ^2^Boston Children’s Hospital, Boston, MA, United States; ^3^Centre for Interdisciplinary Research in Music Media and Technology (CIRMMT), Montréal, QC, Canada; ^4^Department of Information Technology, WAVES Research Group, Ghent University, Ghent, Belgium; ^5^Department of Health and Care, Artevelde University of Applied Sciences, Ghent, Belgium; ^6^School of Information Studies, McGill University, Montréal, QC, Canada

**Keywords:** hearing protection, music performance, auditory health, musical practice, piano, MIDI, flange earplugs, musician wellbeing

## Abstract

Professional musicians are often exposed to high noise levels and thus face the risk of noise-induced hearing loss. Yet, adoption rates for hearing protection among musicians are low. Previous surveys indicate that the chief concern is the effect of hearing protection use on performance. However, few studies have investigated actual changes in performance when wearing hearing protection. We report an experiment investigating differences in pianists’ performance and experience with and without hearing protection. We compare the effect of foam earplugs and musician earplugs, designed to preserve sound quality with a flat frequency response. The analysis revealed that participants performed overall more loudly with the foam earplugs than with the musician earplugs, and in turn performances with the musician earplugs were louder than the open condition, indicating a compensatory effect. However, this effect was stronger for novel excerpts than for familiar excerpts. No effect was observed on dynamic range. Furthermore, we observed an acclimatization effect, whereby the effect of hearing protection use, observed on the first performance, decreased on the second performance. In terms of experience, participants reported changes in coloration, difficulties gauging dynamics and articulation, and increased effort required when performing with hearing protection. These effects were more pronounced when wearing the foam earplugs, and the participants reported finding the musician earplugs more comfortable to wear and play with. In conclusion, hearing protection use affects pianists’ performance particularly in terms of dynamics and their experience more so in terms of coloration. But the effects are less marked for familiar pieces and after repetition, suggesting that pianists can quickly adjust their playing when playing familiar pieces with hearing protection.

## Introduction

Professional musicians are often exposed to noise levels that exceed occupational exposure limits and thus face the risk of noise-induced hearing loss (Thom et al., 2005). While there is some disagreement in the literature as to whether the incidence of noise-induced hearing loss is in fact higher among musicians than among the general population, there is no debate that the risk of hearing loss can be reduced by use of hearing protection. While standard foam earplugs tend to distort the spectral content reaching the wearer, a variety of specialized musician earplugs have been designed with the aim of providing a flatter frequency response to preserve sound quality — an obvious concern for the professional musician. Despite advances in hearing protection technology, however, various studies have found use of hearing protection devices among professional musicians to be low and inconsistent.

## Literature Review

### Musicians’ Use of Hearing Protection

Laitinen (2005) found that only 6% of participants from orchestras in Finland reported consistent use of hearing protection devices, and 35% reported seldom use. A similar study of Danish orchestra musicians (Laitinen and Poulsen, 2008) found consistent use in 15% of participants while 49% used earplugs in both ears only occasionally. A survey of German orchestra musicians (Zander et al., 2008) found occasional use among 38% of participants and of them, 6–15% use custom-molded earplugs. Jansen et al. (2009) found that 36–85% of participants from professional orchestras use some form of hearing protection and of those who do, most report using disposable protectors. A study from Australia (O’Brien et al., 2014) found higher rates of use (64%) than those found in European studies and the authors attribute this finding to what they consider to be a more robust hearing conservation program in Australia. While this does seem to suggest that education programs can play a role in increasing use of hearing protection, it should be noted that O’Brien et al. (2014) compared results to European studies from five to 10 years prior, and that rate of use could now be higher in Europe as well. While these studies indicate a range in rates of use, the results nevertheless confirm low rates of hearing protection use among professional musicians.

### Factors Influencing Use- Perceived Changes in Performance With Hearing Protection

In addition to rate, the questionnaire studies cited above also investigate musicians’ perspectives with regard to the advantages and challenges of hearing protection use. The main barrier to adoption is the potential effect of wearing hearing protection on musical performance. Among instrumentalists, the chief complaint was the inability to assess the sound of their own instrument, followed by difficulties hearing others, leading to problems of balance and intonation in ensemble settings (Laitinen, 2005; Laitinen and Poulsen, 2008; Zander et al., 2008; Jansen et al., 2009; O’Brien et al., 2014; Beach and O’Brien, 2017). In one study of choral singers—who are also exposed to high noise levels—singers report greater difficulty hearing others than hearing themselves when using hearing protection (Cook-Cunningham, 2019).

More specifically, musicians report distortions of timbre and dynamics (Huttunen et al., 2011). These concerns create hesitancy among musicians, who feel that wearing protection could put them at a disadvantage in a highly competitive professional environment. Some users of hearing protection report removing hearing protection for particularly complex passages (Laitinen and Poulsen, 2008) or using hearing protection in one ear only (Laitinen and Poulsen, 2008). The degree of difficulty in playing with hearing protection varies across instruments, where brass (and specifically trumpet) players report the greatest difficulty (see Mead (2012) for an in-depth discussion of trumpet players’ use of hearing protection). Likewise, the rate of use of hearing protection in orchestras is lowest among brass players (Zander et al., 2008).

### Other Factors Influencing Hearing Protection Use

While these studies indicate that the chief barrier is concern over the effect of hearing protection on performance, the fact remains that some musicians do choose to wear hearing protection—and consistently so. What factors compel certain musicians to wear hearing protection despite concerns over performance?

One common finding among these studies is higher rate of hearing protection use among musicians with existing hearing complaints (Laitinen, 2005; Laitinen and Poulsen, 2008; Zander et al., 2008). While it is possible that not all of these hearing complaints are noise-induced, this suggests a lack of preventive use and that musicians are more likely to use hearing protection after a certain degree of damage has already been done. While disheartening on the one hand, this also suggests that musicians are receptive to wearing hearing protection after they have experienced some damage, which can aid in stabilizing their condition and preventing further hearing loss.

Different rates of use are consistently noted among players of different instruments (Laitinen and Poulsen, 2008; Zander et al., 2008; Chesky et al., 2009; Jansen et al., 2009; O’Brien et al., 2014; Beach and O’Brien, 2017). This in large part seems to be related to the fact that some instruments are more difficult to play than others when wearing hearing protection (see discussion above). Additionally, however, Zander et al. (2008) found a positive correlation between use of hearing protection and perception of loudness of one’s own instrument as well as loudness of neighboring instruments (Zander et al., 2008). Highlighting the subjective nature of loudness, Rawool and Buñag (2019) suggest that perception of loudness among musicians—and thus willingness to use hearing protection—may be influenced by cultural background (comparing here Caucasian and Filipino musicians, but playing different instruments). In a series of in-depth interviews with various musicians, Beach and O’Brien (2017) found that student participants expressed more advantages related to the use of earplugs compared to professional and amateur musicians. These findings indicate a possible interaction between individual factors and instrument type in the decision to use hearing protection and warrant further exploration.

### Measured Effects of Changes in Performance With Hearing Protection

While qualitative studies are an important aspect in incorporating musician perspectives into education initiatives and development of technology, another complementary approach is to investigate measurable changes in performance with and without earplugs through acoustic analysis. This approach has been taken in an industry setting when studying the effect of earplugs on speech production but few studies have done so with musician performance.

In the context of speech production, two main effects have been documented: the Lombard effect and the occlusion effect. The Lombard effect is a well-documented phenomenon whereby speakers increase their level of speech relative to increased environmental noise. The occlusion effect is another phenomenon whereby self-generated sounds (speech, chewing, swallowing) are perceived to be of increased loudness when the ears are covered. The ability to adjust speech levels to noise has been shown to be affected by use of hearing protectors whereby the level of speech produced in noise while wearing hearing protectors is lower compared to speech in noise without hearing protection (Howell and Martin, 1975; Tufts and Frank, 2003; Bouserhal et al., 2016). However, this decrease is not observed in speakers wearing hearing protection in quiet conditions (Tufts and Frank, 2003) and Howell and Martin (1975) actually found an increase in level in quiet conditions while wearing hearing protection.

Turning to the effect of hearing protection use on musician performance, a finding analogous to the Lombard effect was reported in choral singers (Cook-Cunningham, 2019) with a 1.30–5.29 dB decrease when singing in a choir with earplugs versus without earplugs. In the same study, recordings of soloist singers were not significantly affected by use of earplugs (< 1 dB change).

With instrumentalists, spectral analyses of musicians’ performances with and without earplugs with various instruments were conducted in solo and ensemble settings (Kozłowski et al., 2011). Specific differences in 1/3-octave bands were observed for different groups of instruments. The greatest change was observed for the trumpeter with a 5–15 dB drop in the high frequency range. By contrast, the spectrum of the clarinetist’s and violinist’s performances were not significantly affected by use of earplugs and the vocalist’s performance changed less than 5 dB. The effect of instrument type as seen in acoustic analysis reaffirms the self-reported findings in the qualitative studies cited above wherein brass players report the most difficulty playing with hearing protection. This effect of instrument type could be explained by the occlusion effect, whereby instrumentalists who produce self-generated sounds, such as singers or brass players, might be susceptible to an increase in loudness perception of their own sounds while wearing earplugs. Consequently, they are likely to sing or play more softly to compensate for the perceived increased loudness. Conversely, instrumentalists whose means of sound production rests outside of the body, such as pianists or string players, should be immune to this effect and might actually play more loudly to compensate for the level attenuation of the sound they produce as it reaches their ear while wearing earplugs.

A third study (Rawool and Buñag, 2019) compared solo performances of musicians playing a wide variety of instruments with and without earplugs in terms of average sound level and dynamic range. The authors cite musicians’ complaint that earplugs hinder their ability to differentiate loud and quiet sounds as a reason to investigate changes in dynamic range. In solo performances, the majority of participants did not significantly alter overall level with earplugs; however, 8–31% of participants played more loudly while wearing earplugs. Differences in dynamic range with and without earplugs were observed in some players, although the degree of change was affected by trial number indicating the possibility of acclimatization. While previous studies support the need for caution when making generalizations across instrument type (e.g., Kozłowski et al., 2011), this finding warrants the investigation of possible acclimatization to hearing protection.

A fourth recent study (MacLeod et al., 2021) investigated the effect of foam earplugs and musician earplugs on pitch perception, using a pitch matching task with music students. Small but significant differences were observed for specific intervals only. Participants’ pitch matching was most accurate in the absence of hearing protection, followed by the musician earplugs condition, and they were least accurate when wearing foam earplugs. The results suggest that musician earplugs may provide valuable protection while minimally affecting pitch perception.

While few in number, the above studies lay the foundation for further research into the measurable changes in musical performance with and without hearing protection. This is not to say that quantitative measures are somehow more valid than subjective experience of performance. For example, if acoustic analysis reveals no difference but musicians report greater effort required to perform with hearing protection, this increased effort could present a barrier to consistent use. Thus, both qualitative and quantitative aspects are needed to better understand the effect of hearing protection on performance.

### Research Questions

With the aim of better understanding the effect of hearing protection use on musical performance, this study has four objectives. First, we investigate pianists’ attitudes toward hearing protection, with a detailed questionnaire on auditory health practices and attitude toward hearing protection. Second, we explore changes in performance with and without hearing protection while controlling for instrument (piano) and performance context (solo). Specifically, we compare the loudness and dynamic range of solo performances recorded by the same pianists under three conditions: without hearing protection, while wearing musician earplugs and while wearing foam earplugs. Third, we investigate acclimatization to hearing protection by comparing first-time and second-time performances. Fourth, we investigate pianists’ experience while performing with and without hearing protection with a short questionnaire administered after each trial.

## Materials and Methods

### Recruitment

Seventeen pianists with more than 10 years of musical training were recruited through the mailing lists of the McGill University Schulich School of Music and the Centre for Interdisciplinary Music, Media and Technology. They received $15 CAD for their participation and took home both pairs of hearing protectors tested.

### Procedure

Participants were first asked to fill out a questionnaire on their auditory health practices. Then, the participants were instructed to play three excerpts under three different hearing conditions: namely, an open condition without hearing protection, while wearing foam earplugs, and while wearing musician earplugs, for a total of 9 trials per participant . Participants performed on a Yamaha upright Disklavier Mark III, which recorded their performance as MIDI data. The order of the hearing conditions was counterbalanced across participants. The order of presentation for the 3 excerpts was fixed, starting with the most familiar, played from memory, to the least familiar, in a sight-reading task (see details below). After each trial, participants filled out a short post-trial questionnaire on their experience. The entire session lasted about an hour.

The excerpts were selected as follows:

• E_0_: Participants were instructed to prepare a short excerpt of their choice, under 2 minutes, before the study. They played this first excerpt from memory. This was designed to provide the participants with maximum familiarity with this excerpt. Selections included pieces by Bach, Mozart, Haydn, Schumann, Schubert, Gershwin, Scriabin, Einaudi and Veloso.
• E_1_: Participants were instructed to sight-read the first 24 bars from the Prelude No. 15, opus 38 by Frederic Chopin. This excerpt was chosen as a common excerpt of medium difficulty from the standard repertoire, and participants were expected to have heard or played it before. This was designed to provide the participants with an intermediate level of familiarity with this excerpt.
• E_2_: The participants were instructed to sight-read the first 17 bars from the Nocturne No. 3 by Francis Poulenc. This excerpt, also of medium difficulty, was chosen because it is a lot less common. This was designed to provide the participants with a low level of familiarity with this excerpt.

**FIGURE 1:**
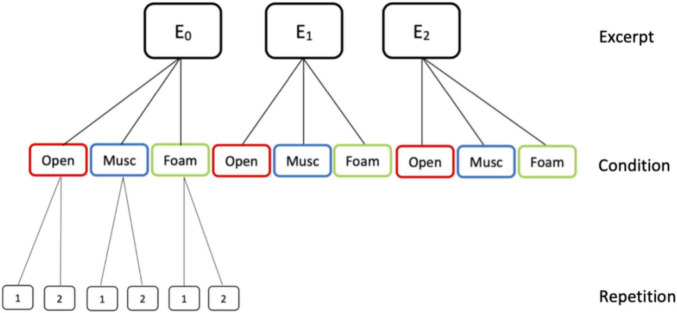
Experimental design. E0 was a highly familiar excerpt played from memory, while E1 (of medium familiarity) and E2 (of low familiarity) were sight-read. All excerpts are performed by all 17 participants in three hearing conditions, namely Open without earplugs, Music while wearing musician earplugs, Foam while wearing foam earplugs. Eleven participants further repeated E0 in all three hearing conditions to investigate acclimatization effects.

Furthermore, a subset of 11 participants was asked to repeat each performance of the familiar E_0_ excerpt under each of the three protection conditions. This was done to investigate a potential acclimatization whereby participants might be less affected by the hearing protection on their second try. The experimental design is represented in Figure 1.

### Hearing Protection

We tested two types of earplugs, namely foam earplugs and musician earplugs. The foam earplugs were standard roll-down slow-recovery foam 3 M 1,100, widely available at a cost of around 1$ per pair, with an advertised Noise Reduction Rating (NRR) of 29 dB (when properly inserted). The musician earplugs used were flange Etymotic ER-20 earplugs, at a cost of $20 per pair, with an advertised attenuation of 20 dB but an official NRR of 12 dB. Frequency-specific attenuation, as provided by the manufacturers, as shown in Table 1. It should be noted that there might be substantial differences between the attenuation advertised and measured on individual users, particular in lower frequencies, because of leakage due to suboptimal placement. For foam earplugs specifically, previous studies measured NRRs around 25 dB (instead of 29) for unsupervised use and observed large inter-individual differences (Berger, 2013; Copelli et al., 2021). Given the stronger and non-uniform sound level attenuation of the foam earplugs, we hypothesized a stronger effect on performance with foam earplugs than with musician earplugs.

**TABLE 1:** Frequency-specific attenuation and Noise Reduction Rating of the foam and musician earplugs as advertised by the manufacturers.

Frequency (Hz)		125	1,000	4,000	8,000	NRR
Attenuation (dB)	Musician earplug	19.2	21.2	20.7	23.6	12
	Foam earplug	20.3	26.5	32.3	41.1	29

### Questionnaires

A general auditory health questionnaire was administered when participants first arrived. The questionnaire consisted of 31 Likert scales and 3 open-ended questions. It was adapted to the musical setting from a previous survey with industry workers (Bockstael et al., 2013) and translated from Dutch. The questionnaire was structured into four sections related to (1) Attitudes towards hearing protection, (2) Auditory health issues, (3) Use of hearing protection, (4) Demographics and musical training. In addition, after completing each condition involving the use of hearing protection, participants filled out a post-trial questionnaire related to their experience performing. The questionnaire consisted of an open-ended question about the perceived sound quality followed by 12 Likert scales on their experience in terms of perceived benefits and disadvantages of hearing protectors, intention to use when practicing and preferences. The full questionnaires are available in the Supplementary Material.

## Results

### Participants

Seventeen music students (4 women, 12 men and 1 other), all currently enrolled at the Schulich School of Music of McGill University, participated in the experiment. They were aged 18 to 37 (mean age 24). Twelve studied piano performance (mostly at the graduate level), either classical or jazz, while the remaining 5 played piano as their secondary instrument. They reported an average of 17 years of musical training and 3.4 hours of daily practice. All participants had prepared a short excerpt to play from memory (E_0_), all reported being somewhat familiar with E_1_ and all reported never having played E_2_ before.

### Auditory Health Questionnaire

All participants reported some concern over their auditory health, and 12 participants (out of 17) reported taking measures to protect their auditory health, such as moving away from loud sounds, avoiding loud live music, and limiting headphone volume. Seven participants reported auditory health issues, specifically tinnitus (*N* = 4), hypersensitivity to loud sounds (*N* = 3), pain related to noise exposure (*N* = 1), and some form of hearing loss (*N* = 1). Yet only 9 (out of 17) participants reported using hearing protection, but only very occasionally and mostly when studying (*N* = 6), attending events or parties with amplified music (*N* = 6), being exposed to loud noises (e.g., construction) (*N* = 6), or sleeping (*N* = 1). Four participants reported using hearing protection in the context of group rehearsals while one other participant reported using it for individual practices but not for group practices. While most participants (14 out of 17) reported that both their own instrument and others’ instruments were loud, they also reported that most musicians did not wear hearing protection, and that playing music with hearing protection was less fun. Furthermore, 13 found hearing protection uncomfortable, and five participants reported being worried that wearing hearing protection would make them look unprofessional.

### Performance Data Analysis

**FIGURE 2:**
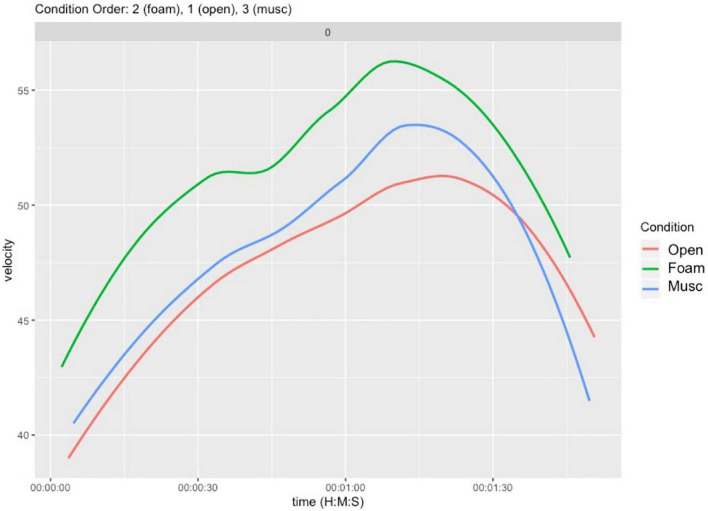
Sample data from a participant performing a familiar excerpt E_0_ in the three hearing conditions (Open, Musician earplugs, Foam earplugs).

From each participant’s MIDI data, we extracted individual note velocity, used as a measure of loudness in previous studies (Lazarov et al., 2019 *inter alios*), for each of the three excerpts in each of three conditions. We represented velocity over time over the entire duration of each excerpt for each trial. An example of this representation is shown in Figure 2. The following analyses rely on the mean velocity per excerpt and condition, collapsing over all participants.

### Effect of Hearing Protection on Loudness

To investigate the effect of hearing protection, we only considered the first performance of each excerpt in each condition (*N* = 68,008 notes), excluding repeated excerpts. A two-way repeated measures ANOVA was performed to evaluate the effect of the hearing protection condition on loudness over three different excerpts. There were significant effects of condition [*F*(2,32) = 130.83, *p* < 0.001] and excerpt [*F*(2,32) = 39.33, *p* < 0.001] as well as a statistically significant interaction between condition and excerpt on velocity [ *F*(4,64) = 49.98, *p* < 0.05]. Therefore, the effect of the hearing protection condition was analyzed separately for each of the three excerpts using one-way repeated ANOVAs for each excerpt with condition as a factor. The effect of condition was significant for all three excerpts [*F*(2,32) = 27.91, *p* < 0.001 for E_0_, *F*(2,32) = 36.96, *p* < 0.001 for E_1_, and *F*(2,32) = 95.43, *p* < 0.001 for E_2_. Post-hoc tests (Tukey HSD with adjusted p-values) revealed significant differences between all pairwise combinations of the three conditions, for each of the three excerpts (*p* < 0.001).

**FIGURE 3:**
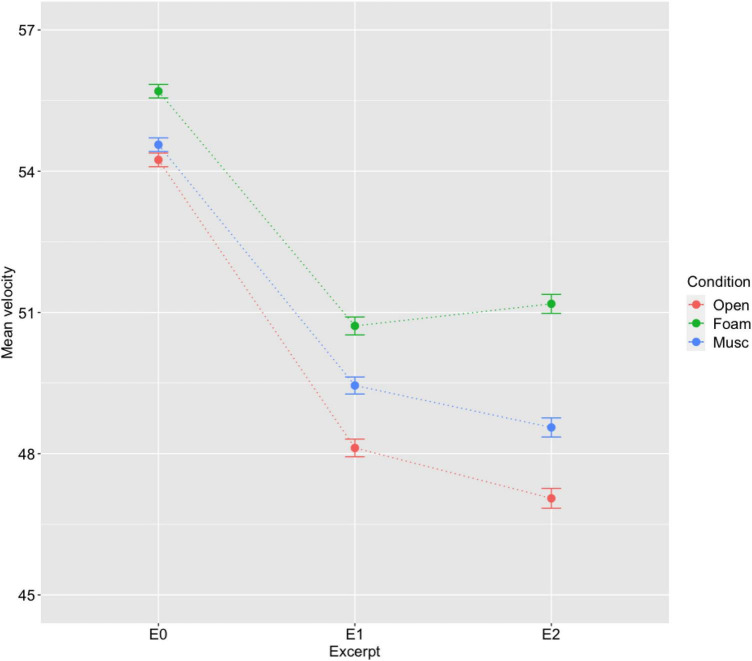
Mean velocity and standard error by excerpt and hearing condition (all 17 participants). Significant differences across hearing conditions (Open, Musician earplugs, Foam earplugs) were observed for each of the three excerpts.

The results shown in Figure 3 indicate that participants performed more loudly while wearing earplugs; that is, they compensated for the sound attenuation of the hearing protection by playing more loudly. As hypothesized, the differences were more marked with the foam earplugs than with the musician earplugs. Specifically, participants played at the softest level in the open condition, followed by the musician earplugs condition, and at the loudest level in the foam earplugs condition. These differences across conditions were significant for all three excerpts, but were less marked for the familiar excerpt E_0_ than for novel excerpts E_1_ and E_2_. The differences in mean MIDI velocities across conditions were 1.46 for E_0_, 2.59 for E_1_ and 4.13 for E_2_ for MIDI velocities in the medium to low range (47 to 55). According to relationships between MIDI velocity (ranging from 20 to 100) and sound level (measured at 3 ft) established by Repp (1997)^1^, these mean level difference across conditions can be estimated under 1 dB for E_0_ (which would not be perceptible), and around 1 dB for E_1_ (which would be just perceptible) and above 1 dB for E_2_ (which should be perceptible).

### Effect of Hearing Protection on Dynamic Range

**FIGURE 4:**
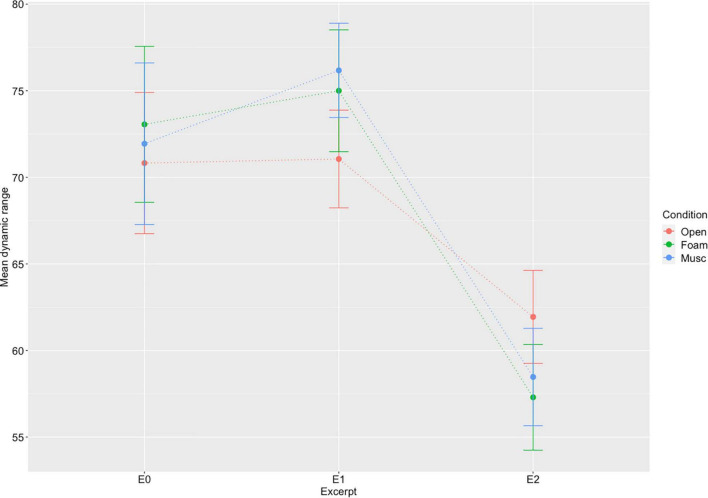
Mean dynamic range (max-min velocity) and standard error by excerpt and hearing condition (Open, Musician earplugs, Foam earplugs) (all 17 participants). No effect of hearing condition was observed.

To further investigate the effect of hearing protection on performance, we evaluated the effect on dynamic range, defined as the difference between the loudest and softest note for each trial. A two-way repeated measures ANOVA was performed to evaluate the effect of hearing protection condition on dynamic range over three different excerpts. A significant main effect of excerpt was observed [*F*(2,32) = 15.58, *p* < 0.001] but no other effects were observed. The results, shown in Figure 4, indicate that dynamic ranges do not vary significantly across hearing condition. A previous study by Rawool and Buñag (2019) operationalized 2 different measures of dynamic range, namely (max-min) and (max-mean). We therefore replicated the analysis for the second measure of dynamic range and obtained similar results; that is, no effect of hearing condition on either measure of dynamic range was observed.

### Effect of Acclimatization

To investigate acclimatization, we only considered the familiar excerpt (E0) which was performed twice in each condition by 11 participants (*N* = 40,045 notes). A two-way repeated measure ANOVA revealed significant effects of condition [*F*(2,32) = 110.56, *p* < 0.001] and repetition [*F*(2,32) = 1,109.78, *p* < 0.001] as well as an interaction effect of condition × repetition on note velocity [*F*(4,64) = 22.61, *p* < 0.001].

**FIGURE 5:**
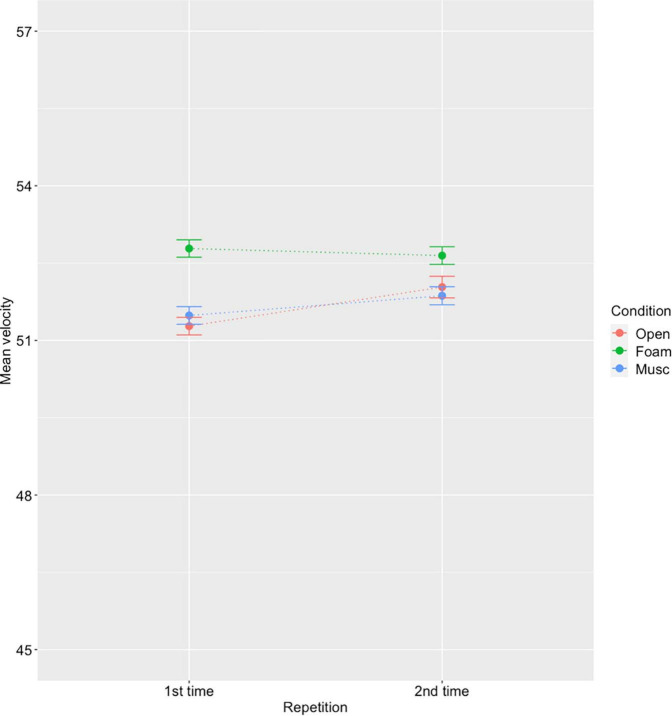
Mean velocity and standard error by repetition and hearing condition for the familiar excerpt E_0_ (for 11 participants). While there was a significant effect of condition on the 1*^st^*- time performance, no significant differences were observed between the open and hearing protection conditions on the 2*^nd^*- time performance, indicating an acclimatization effect.

Therefore, the effect of the hearing protection condition was analyzed separately using one-way repeated ANOVAs for each repetition with condition as a factor. The effect of condition was significant for both repetition conditions [*F*(2,32) = 27.91, *p* < 0.001 for first time, *F*(2,32) = 21.4, *p* < 0.001 for the second time]. Further analyzing differences for the first-time performance, post-hoc tests (Tukey HSD with adjusted *p*-values) revealed significant differences between Open and Foam conditions as well as between Foam and Musician conditions (both *p* < 0.001) but no significant difference between Open and Musician conditions (*p* = 0.25). For the second performance, post-hoc tests revealed a significant difference between Foam and Musician conditions (*p* = 0.004) but no significant differences between Open and Musician (*p* = 0.8) or between Foam and Open conditions (*p* = 0.06). In other words, on the second performance, no significant differences were observed between the open condition and the two hearing protection conditions. Indeed, as shown in Figure 5, the differences across hearing conditions are less marked for the second time than for the first time. This indicates an acclimatization effect over only a few minutes. While velocities remain unchanged in the Foam condition, we observed an increase in velocity after repetition in the Open and Musician conditions, which could be attributed to increased confidence and familiarity with the instrument.

### Effect of Hearing Protection on Experience

**FIGURE 6:**
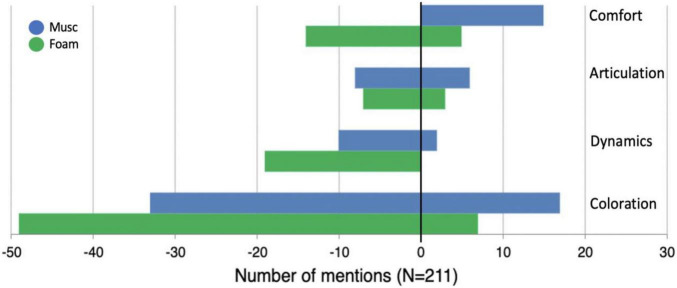
Mentions of sound quality descriptors for the foam and musician earplugs conditions. Mentions with a negative connotation are displayed with negative values; mentions with a positive connotation with positive values. The number of mentions collected is the absolute value.

The effect on experience was investigated through the post-trial questionnaire analysis. The free-form answers to the sound quality question were grouped into four broad themes using an inductive content analysis. Specifically, the first and last author first segmented free-format responses into individual mentions and independently categorized them into themes emerging from the constant comparison method (Glaser and Strauss, 1967). They then discussed their categorization and harmonized the themes accordingly. The themes were related to Coloration (106 mentions), Comfort (41 mentions), dynamics (40 mentions), and articulation (24 mentions). Each mention was categorized as either positive or negative based on the connotation inferred from the free-form answers. The distribution of positive and negative mentions for each of the 4 themes is represented in Figure 6. The majority of mentions were related to coloration, with participants most often reporting a dull and muffled quality of sound when wearing earplugs, with more negative mentions for foam earplugs than for musician earplugs. Comfort description referred to how comfortable participants felt while playing with earplugs, as well as difficulties encountered, such as spatial disorientation and additional effort needed. In terms of dynamics, participants reported that their playing felt quiet. They further expressed difficulties gauging dynamics and trouble hearing certain ranges (both piano and forte). In terms of articulation, they reported the need to articulate more, issues with attacks, difficulties gauging the amount of pedal needed.

Overall, the difficulties and discomfort level were higher when wearing foam earplugs than musician earplugs, as can be seen from the distribution of negative and positive mentions.

The analysis of the Likert scales was in line with the qualitative results. Indeed, the overall satisfaction level was significantly lower for foam earplugs (2.6 out of 5) than musician earplugs (3.4 out of 5) (*p* < 0.0001). Similarly, the sound was perceived as significantly boomier (2.6 out of 5) with foam earplugs than with musician earplugs (1.9 out of 5) (*p* < 0.001). In terms of overall experience, participants reported a strong preference for the open conditions (4.5. out of 5) and their intention to use or buy either type of hearing protection was low (2.1 out of 5).

## Discussion

### Attitudes and Experience

While all participants expressed concerns over their auditory health, only half of them reported using hearing protection, and mostly did so outside of a musical context. Possible explanations for limited use of hearing protection include the reported discomfort and decreased enjoyment when practicing with hearing protection, as well as the perceived low adoption rates of hearing protection among colleagues.

In terms of actual experience while playing with earplugs, pianists reported changes in coloration (more muffled sound), difficulties gauging dynamics and articulation, and additional effort required. These disadvantages could be even more problematic in an ensemble context, particularly to hear subtle cues (e.g., breathing cues) and finer adjustments needed to achieve ensemble blend.

Although our participants generally disliked playing with earplugs, they were more comfortable with the musician earplugs than with the foam earplugs. Being aware that one type of protection was designed for musicians might have affected their perception. It is understandable that unacclimatized musicians would feel discomfort when first playing with earplugs, but it is encouraging that their experience was more positive when wearing musician earplugs. This provides support for the use of musician earplugs and encourages further technological initiatives aimed at preserving musicians’ auditory health.

Compared with previous studies on hearing protection in industry workplaces (see Bockstael et al., 2013 for a review), our participants were more aware of the potential risks of noise exposure than industry workers. Yet, they did not seem to evaluate the risk as high in the context of musical practice, as most of them only used hearing protection outside of musical activities. It should be noted that our participants are pianists, and as such may be less exposed to harmful sound levels than orchestral musicians, given that they are more likely to play alone or in small ensembles. However, studies in industry caution about the underestimation of the risk of noise-induced hearing loss in environments where sound levels are comparatively lower but still potentially harmful. Furthermore, studies on occupational noise highlight the role of the working environment. Specifically, safety policies contribute to higher adoption rates (Bockstael et al., 2013). This suggests that institutional encouragement about hearing conservation for musicians, as part of a curriculum or in professional ensembles, might foster more widespread use of hearing protection .

### Performance

Our experiment revealed that participants played more loudly when wearing hearing protection, and this effect was stronger when they wore foam earplugs than musician earplugs. This indicates a compensatory effect whereby participants increased the loudness of their playing to compensate for the level attenuation caused by the earplugs. This is further supported by the fact that participants played at the loudest level with the foam earplugs, which have greater attenuation. However, this is in contradiction with previous studies where musicians, usually singers or brass players, instead played more quietly when wearing hearing protection (Kozłowski et al., 2011). This discrepancy can be explained by the occlusion effect, namely that musicians who produce self-generated sounds perceive an increased level of their own sound when wearing earplugs. This perceived increase in sound level would in turn incite singers and brass players to compensate by playing less loudly. However, the pianists in our study perceived instead a decrease in sound level when wearing protection. This can be attributed to the external sound production on the piano (as opposed to an embodied sound production in speech, singing or brass instruments). The level of the external sound reaching the ear is attenuated by the earplugs, and there is no (or very little) bone-conduction transmission. Consequently, pianists compensate in the opposite direction, playing more loudly to make up for the diminished auditory feedback. These differences highlight the influence of instrument type on the effects of hearing protection on performance. Particularly, singers and wind instrumentalists who produce sounds with their mouths, lips and vocal cords may be more susceptible to being affected by hearing protection. Indeed, these musicians need to adjust not only for the external sound level attenuation caused by the earplugs but also for the occlusion effect, resulting in conflicting cues likely to affect their perception of timbre in addition to loudness. This could explain why previous studies found brass players most reluctant to wear hearing protection, even though they are usually exposed to the highest sound levels in orchestral settings (Zander et al., 2008). Pianists, string players, and other musicians who produce sound externally may indeed have an easier time adjusting to wearing hearing protection because the occlusion effect is less, if at all, prevalent.

As previous research (Rawool and Buñag, 2019) had observed an effect of hearing protection on the dynamic range of music performance, we also investigated this phenomenon but found no effect of hearing condition on dynamic range. This could be attributed to two main differences: (1) the instrument types (strings, winds and piano in Rawool and Bunag as opposed to pianists only in this study), (2) the analysis method (audio recording analysis in Rawool and Bunag versus MIDI velocity data in this study).

Our study further reveals the importance of another factor affecting hearing protection use, namely the degree of familiarity with the music performed and/or the task at hand. Indeed, in our experiment with pianists, the effects of wearing hearing protection were less marked when playing familiar excerpts from memory than when sight-reading novel excerpts. This finding suggests that the compensatory effect that caused the participants to play more loudly with hearing protection was moderated by familiarity with the piece they were playing. This could be attributed to the fact that pianists performing familiar pieces play more confidently, rely less on the auditory feedback and more on muscle memory than when sight-reading. But this also suggests that musicians working on familiar pieces, as in the context of practicing standard repertoire, could be somewhat protected from the negative effects of wearing hearing protection. Since some protection is better than none, this finding could encourage musicians to wear hearing protection in musical contexts where they feel more at ease, even if they remain reluctant to do so in higher stakes environments. Finally, the lesser effect of hearing condition for the familiar excerpt could also be related to the fact that it was played more loudly than less familiar excerpts, at a level at which hearing protection might be less efficient.

Our findings also suggest an acclimatization effect whereby the differences in loudness across hearing protection conditions which were observed during the first performance were no longer significant on the second performance. In other words, the compensatory effect disappeared on the first repetition, after only a few minutes of wearing hearing protection. This suggests that musicians could have the ability to quickly adapt to wearing earplugs, which could lead to a reduction of the negative effects of hearing protection after prolonged use. Furthermore, if musicians can easily adapt their playing when wearing hearing protection after a short training period (as they do when playing with a practice mute), they may be able to use earplugs in certain conditions (e.g., when practicing long hours in small reverberant practice rooms) and opt out for other situations (e.g., when performing in a large concert venue). Alternatively, in ensemble settings where they might be exposed to higher levels, they could practice (alone) with hearing protection ahead of time, in order to prepare for group performances where protection is most needed. Future research is needed to determine the extent to which musicians can learn to adjust to different hearing protection conditions.

### Limitations and Future Directions

Our investigation was limited to a particular instrument (piano) and performance context (solo). Given that the effects of hearing protection in performance differ across instrument types, the results may not generalize to other musical contexts. Furthermore, there are reasons to believe that different instrument types may be affected very differently by hearing protection use, particularly because of the occlusion effect which only applies to embodied sound generation. Conducting similar research with a wide range of instrument types, while accounting for the method of sound production, would allow us to reconcile conflicting research findings across studies with different instruments.

Additionally, performance in an ensemble context is likely to be more affected by hearing protection use. Indeed, the Lombard effect, which predicts that speakers raise their voice in louder environments, could apply to music performance in ensemble contexts. In other words, performers wearing earplugs would play softer because of the sound level attenuation of the surrounding environment. This might counterbalance or at least reduce the compensatory effect observed in this study, where solo musicians played more loudly when wearing earplugs.

Future research is also needed to investigate whether the beneficial effects of familiarity and acclimatization also apply in the context of ensemble playing. Musicians are at a greater risk of noise-induced hearing loss in ensemble contexts, yet they are also more reluctant to use hearing protection in such contexts. Testing these effects of familiarity and acclimatization in ensemble settings could encourage musicians to persevere through the initial challenges encountered when wearing hearing protection. This could contribute to raising the rate of use of hearing protection devices in orchestral contexts.

In their responses, our participants very clearly favored the musician earplugs over the foam ones. It would be important to examine whether musicians would react even more favorably to custom-made earplugs that fit the morphology of their ear with a pre-specified attenuation level. This could encourage hesitant musicians to invest in such devices. Additionally, the attenuation of the earplugs used in this study (in the 20–30 dB range) is perhaps too large for certain musical contexts, particularly for smaller ensembles and quieter instrument types. Certain musicians may get sufficient hearing protection with lower attenuation earplugs while suffering less from the negative effects that come with wearing earplugs. Having a range of attenuation options for musicians to choose from, along with research to inform that choice, could also go a long way to encourage more widespread use.

Another promising path towards the wide-spread adoption of hearing protection devices lies in a new trend of musicians considering earplugs as potential practicing tools. Indeed, a previous study reported on the use of earplugs as a “performance enhancer”; that is, a tool used by performers to distance themselves from their sound in order to mimic the sound environment of larger concert halls in their practice rooms (Beach and O’Brien, 2017). Such approaches could encourage hearing protection use in a variety of contexts and provide musicians with creative ways to experiment and get acclimatized to wearing hearing protection.

Overall, this study contributes to the small yet growing body of literature aiming to increase understanding of the factors influencing hearing protection use among musicians. While we observed a compensatory effect of wearing hearing protection on overall loudness, this effect disappeared after only one repetition, and was less pronounced with familiar excerpts and with musician earplugs. These results suggest that musicians can quickly learn to adjust their playing when wearing earplugs, even if their experience might be negatively affected. Our findings highlight the need for education campaigns about the effects of hearing protection so that musicians can balance the pros and cons to prevent noise-induced hearing loss without compromising the quality of their performance.

## Data Availability Statement

The raw data supporting the conclusions of this article will be made available by the authors, without undue reservation.

## Ethics Statement

The studies involving human participants were reviewed and approved by McGill Research Ethics Board II. The patients/participants provided their written informed consent to participate in this study.

## Author Contributions

AB, CG, and SB conceived and planned the experiment. EB analyzed the data under the guidance of CG and AB. EB wrote the manuscript with support from CG, AB, and SB. All authors contributed to the article and approved the submitted version.

## Conflict of Interest

The authors declare that the research was conducted in the absence of any commercial or financial relationships that could be construed as a potential conflict of interest.

## Publisher’s Note

All claims expressed in this article are solely those of the authors and do not necessarily represent those of their affiliated organizations, or those of the publisher, the editors and the reviewers. Any product that may be evaluated in this article, or claim that may be made by its manufacturer, is not guaranteed or endorsed by the publisher.

## Footnotes

1 Using a Yamaha Disklavier Mark II grand piano recorded at 3 feet from the curved rim of the frame.

## Supplementary Material

Supplementary Datasheet 1 Auditory health questionnaire.

Supplementary Datasheet 2 Post-trial questionnaire.
