# Supplementary material for: Effect of Hearing Protection Use on Pianists’ Performance and Experience: Comparing Foam and Musician Earplugs
Source: Front Psychol. 2022 Jul 12;13:886861. doi: 10.3389/fpsyg.2022.886861 (PMC9315358; doi:10.3389/fpsyg.2022.886861)
Supplement: Supplementary Datasheet 2 — Post-trial questionnaire. [file Data_Sheet_2.pdf]

Post-questionnaire for hearing protection experiment with musicians, June2019

The following is questionnaire for a study on hearing protection with musicians. It is designed to be answered after performing a piece of music wearing hearing protection.

This study is conducted under the supervision of Profs. Catherine Guastavino (catherine.guastavino@mcgill.ca, McGill School of Information studies) and Annelies Bockstael (Université de Montreal École d'orthophonie et d'audiologie).

Thank you for your participation.

There are 5 questions in this survey

Test Condition Responses

The following questions are designed to be answered after performing a piece of music while wearing hearing protection.

[]Please select the type of earplugs you just used: *

Choose one of the following answers

Please choose **only one** of the following:

- ☐ Foam earplugs
- ☐ Musician earplugs

[]I just played the following piece: *

Choose one of the following answers

Please choose **only one** of the following:

- ☐ My own piece
- ☐ Excerpt 1
- ☐ Excerpt 2

[]To the best of your ability, describe the sound quality you experienced while wearing these hearing protectors (e.g. dull, clear, etc). *

Please write your answer here:

[]On a scale of 1-5, please indicate the extent to which you agree with the following statements (1=Strongly disagree, 5=Strongly agree) *

Please choose the appropriate response for each item:

	1 (Strongly Disagree)	2	3	4	5 (Strongly Agree)
**Regardless of the price, I would buy (or have bought) these hearing protectors to wear while playing music.**	☐	☐	☐	☐	☐
**In general, I am satisfied with the hearing protectors I just used.**	☐	☐	☐	☐	☐
**I suppose the music would have sounded just as good without hearing protectors.**	☐	☐	☐	☐	☐
**These hearing protectors are very discrete and are hardly noticeable when I wear them.**	☐	☐	☐	☐	☐
**The hearing protectors make the music sound too quiet.**	☐	☐	☐	☐	☐
**The hearing protectors make the music sound boomy, like I'm in a tunnel.**	☐	☐	☐	☐	☐
**I could wear these hearing protectors comfortably during the entire performance.**	☐	☐	☐	☐	☐
**I would practice regularly with these hearing protectors.**	☐	☐	☐	☐	☐
**Once I fitted the hearing protectors, they stayed in place and I had the feeling they fit perfectly.**	☐	☐	☐	☐	☐
**These hearing protectors provide me with sufficient protection.**	☐	☐	☐	☐	☐
**I feel ridiculous with these hearing protectors.**	☐	☐	☐	☐	☐
**I would have preferred to play without the hearing protectors.**	☐	☐	☐	☐	☐

[]Please comment if any of the above questions were unclear or did not apply to you:

Please write your answer here:

Submit your survey.
Thank you for completing this survey.
