# Supplementary material for: Effect of Hearing Protection Use on Pianists’ Performance and Experience: Comparing Foam and Musician Earplugs
Source: Front Psychol. 2022 Jul 12;13:886861. doi: 10.3389/fpsyg.2022.886861 (PMC9315358; doi:10.3389/fpsyg.2022.886861)
Supplement: Supplementary Datasheet 1 — Auditory health questionnaire. [file Data_Sheet_1.pdf]

Pre-test questionnaire - hearing protection with musicians, June 2019

The following is a pilot questionnaire for a study on hearing protection with musicians. Please fill it out at your convenience - it should take about ten minutes. Your responses will remain anonymous.

This study is conducted under the supervision of Profs. Catherine Guastavino (catherine.guastavino@mcgill.ca, McGill School of Information studies) and Annelies Bockstael (annelies.bockstael@umontreal.ca, Université de Montreal École d'orthophonie et d'audiologie).

Thank you for your participation.

There are 31 questions in this survey

Use of Hearing Protection

[]

Below you will find some statements related to your use of hearing protection and attitudes toward risk of hearing loss. On a scale of 1 to 5, indicate the degree to which you agree or disagree with each statement (1=Strongly disagree, 5=Strongly agree). *

Please choose the appropriate response for each item:

	1 (Strongly disagree)	2	3	4	5 (Strongly Agree)	NA / Don't know
I look cool when I'm not wearing hearing protection.						
Listening to music while wearing hearing protection is less fun.						
Wearing hearing protection makes me look unprofessional.						
Playing music while wearing hearing protection is less fun.						
Hearing loss would have a large effect on my professional life.						
I know where I can get good hearing protectors.						
I haven't found any hearing protectors that are comfortable and fit me well.						
It is my own responsibility to prevent hearing damage.						
I would use hearing protection if it was given to me for free.						
I am not at any risk for noise-induced hearing loss.						
My instrument is loud.						
It's important to be able to feel the music when I listen to it (it has to be loud enough).						
Hearing loss would have a large effect on my social life.						
Most of my fellow musicians wear hearing protection while rehearsing.						
Most of my fellow musicians wear hearing protection while performing.						
Most of my fellow musicians wear hearing protection while attending concerts with loud music.						
There are good reasons not to wear hearing protection while rehearsing.						
There are good reasons not to wear hearing protection while performing.						
There are good reasons not to wear hearing protection while attending concerts with amplified music.						
When playing in groups/ensembles, others' instruments are loud.						

[]*Please comment if you felt that any of the above questions were unclear or do not apply to you:*

Please write your answer here:

[]Which of the following hearing issue(s) have you experienced in the past year? Check all that apply and add comments where applicable. *

Comment only when you choose an answer.

Please choose all that apply and provide a comment:

- None
- Ringing/tinnitus
- Hearing loss
- Hypersensitivity to loud sounds
- Pain related to noise exposure
- Difficulty understanding speech in noise
- Other:

[]When do you experience ringing/tinnitus? Comment when applicable. *

Only answer this question if the following conditions are met:
Answer was at question '3 [G1Q00003]' (Which of the following hearing issue(s) have you experienced in the past year? Check all that apply and add comments where applicable.)

Comment only when you choose an answer.

Please choose all that apply and provide a comment:

- Sometimes after being exposed to loud sounds.
- Always after being exposed to loud sounds.
- Always.
- I was born with these issues.

[]When do you experience hearing loss? Comment when applicable. *

Only answer this question if the following conditions are met:
Answer was at question '3 [G1Q00003]' (Which of the following hearing issue(s) have you experienced in the past year? Check all that apply and add comments where applicable.)

Comment only when you choose an answer.

Please choose all that apply and provide a comment:

- Sometimes after being exposed to loud sounds.
- Always after being exposed to loud sounds.
- Always.
- I was born with these issues.

[]When do you experience hypersensitivity to loud sounds? Comment when applicable. *

Only answer this question if the following conditions are met:
Answer was at question '3 [G1Q00003]' (Which of the following hearing issue(s) have you experienced in the past year? Check all that apply and add comments where applicable.)

Comment only when you choose an answer.

Please choose all that apply and provide a comment:

- Sometimes after being exposed to loud sounds.
- Always after being exposed to loud sounds.
- Always.
- I was born with these issues.

[]When do you experience pain related to noise exposure? Comment when applicable. *

Only answer this question if the following conditions are met:
Answer was at question '3 [G1Q00003]' (Which of the following hearing issue(s) have you experienced in the past year? Check all that apply and add comments where applicable.)

Comment only when you choose an answer.

Please choose all that apply and provide a comment:

- Sometimes after being exposed to loud sounds.
- Always after being exposed to loud sounds.
- Always.
- I was born with these issues.

[]When do you experience difficulty understanding speech in noisy conditions? Comment when applicable. *

Only answer this question if the following conditions are met:
Answer was at question '3 [G1Q00003]' (Which of the following hearing issue(s) have you experienced in the past year? Check all that apply and add comments where applicable.)

Comment only when you choose an answer.

Please choose all that apply and provide a comment:

- Sometimes after being exposed to loud sounds.
- Always after being exposed to loud sounds.
- Always.
- I was born with these issues.

[]To what extent do you worry about the possibility of developing hearing problems? *

Choose one of the following answers

Please choose **only one** of the following:

- Very much and I try to prevent it.
- A little and I adapt my behavior.
- A little but I don't do anything to prevent it.
- Not worried at all.
- I don't think about it.

[]Indicate on a scale of 1 to 5 to what extent you take the following measures to protect your hearing (1=Never, 5=Always). *

Please choose the appropriate response for each item:

	1 (Never)	2	3	4	5 (Always)
Wearing hearing protection					
Moving further away from sources of loud music					
Avoiding places where loud music is playing					
Limiting the volume of my personal music device					
Checking the sound level in the environment (using an app, etc.)					
I have modified my instrument or my practice environment to limit exposure to loud sounds while I am practicing					

[]Which of the following types of hearing protection have you tried when exposed to noise or music? Check all that apply and comment when applicable. *

Comment only when you choose an answer.

Please choose all that apply and provide a comment:

- I never wear hearing protection
- Disposable earplugs
- Pre-molded flange earplugs
- Custom-made earplugs
- Earmuffs
- Other:

[]

Do the flange earplugs you use have features specifically designed for musicians?

*

Only answer this question if the following conditions are met:
Answer was at question '11 [G1Q00011]' (Which of the following types of hearing protection have you tried when exposed to noise or music? Check all that apply and comment when applicable.)

Please choose the appropriate response for each item:

Yes	Uncertain	No

[]

Do the custom-made earplugs you use have features specifically designed for musicians?

*

Only answer this question if the following conditions are met:
Answer was at question '11 [G1Q00011]' (Which of the following types of hearing protection have you tried when exposed to noise or music? Check all that apply and comment when applicable.)

Please choose the appropriate response for each item:

Yes	Uncertain	No

[]

Do the earmuffs you use have features specifically designed for musicians?

*

Only answer this question if the following conditions are met:
Answer was at question '11 [G1Q00011]' (Which of the following types of hearing protection have you tried when exposed to noise or music? Check all that apply and comment when applicable.)

Please choose the appropriate response for each item:

Yes	Uncertain	No

[]Why did you start using hearing protection? Check all that apply and comment when applicable. *

Only answer this question if the following conditions are met:
Answer was at question '11 [G1Q00011]' (Which of the following types of hearing protection have you tried when exposed to noise or music? Check all that apply and comment when applicable.)

Comment only when you choose an answer.

Please choose all that apply and provide a comment:

- Because I want to protect myself.
- Advice from parents/teachers/friends/medical professional/other…
- Because my fellow musicians use them.
- Because I already have hearing damage.
- Other:

[]Indicate on a scale of 1 to 5 how often you wear hearing protection in the following conditions (1=Never, 5=Always). *

Only answer this question if the following conditions are met:
Answer was at question '11 [G1Q00011]' (Which of the following types of hearing protection have you tried when exposed to noise or music? Check all that apply and comment when applicable.)

Please choose the appropriate response for each item:

	1 (Never)	2	3	4	5 (Always)	NA
During solo rehearsals						
While performing						
When trying to focus in noisy environments (e.g. studying)						
When sleeping						
During group rehearsals						
When attending a concert with amplified music.						
When attending a concert without amplified music.						
When going to bars/parties where there is amplified music.						
When exposed to loud noise (e.g. construction)						

[]Below you will find some statements related to your experience using hearing protection. On a scale of 1 to 5, indicate the degree to which you agree or disagree with each statement (1=Strongly disagree, 5=Strongly agree). *

Only answer this question if the following conditions are met:
Answer was at question '11 [G1Q00011]' (Which of the following types of hearing protection have you tried when exposed to noise or music? Check all that apply and comment when applicable.)

Please choose the appropriate response for each item:

	1 (Strongly Disagree)	2	3	4	5 (Strongly Agree)	NA / Don't know
Hearing protectors fit comfortably.						
I wear hearing protection because my fellow musicians do too.						
When I wear hearing protection while playing music, I remove them from time to time.						
I wear hearing protection while playing or listening to music because I am aware of the potential for hearing damage.						
With hearing protection, it's more difficult to play music alone.						
With hearing protection, it's more difficult to play music in small groups.						
With hearing protection, it's more difficult to play music in large ensembles.						
When wearing hearing protection and playing with other musicians, it is more difficult to interact with each other.						
I know when I should replace my hearing protectors with new ones.						

[]Please comment if any of the above statements were unclear to you:

Only answer this question if the following conditions are met:
Answer was at question '11 [G1Q00011]' (Which of the following types of hearing protection have you tried when exposed to noise or music? Check all that apply and comment when applicable.)

Please write your answer here:

[]Have you ever heard of any initiatives aimed at preventing noise-induced hearing loss? If so, please list:

Please write your answer here:

Background Questions

[]What is your profession? *

Please write your answer here:

[]If you are a student, please indicate your major:

Please write your answer here:

[]If you are a student, indicate your institution:

Check all that apply

Please choose **all** that apply:

- Rather not say
- Institution::

[]What is your main instrument? *

Please write your answer here:

[]Number of years of musical training:

Only numbers may be entered in this field.

Please write your answer here:

[]On average over the past year, how many hours per day do you practice? *

Only numbers may be entered in this field.

Please write your answer here:

[]What types of groups/ensembles do you play in? Check all that apply and comment when applicable. *

Comment only when you choose an answer.

Please choose all that apply and provide a comment:

- solo
- accompanist
- small ensemble
- large ensemble
- Other:

[]Do you play any other instruments? If so, please list: *

Please write your answer here:

[]On a scale of 1-5, please indicate the extent to which you agree to the following statements. (1=Strongly disagree, 5=Strongly agree). *

Please choose the appropriate response for each item:

	1 (Strongly Disagree)	2	3	4	5 (Strongly Agree)
I am sensitive to noise					
I find it hard to relax in a place that's noisy					
I get mad at people who make noise that keeps me from falling asleep or getting work done					
I get annoyed when my neighbors are noisy					
I get used to most noises without much difficulty					
I have modified my instrument or practice environment to limit exposure to loud sounds while I am practicing					

[]Age:

Only numbers may be entered in this field.

Please write your answer here:

[]Gender *

Choose one of the following answers

Please choose **only one** of the following:

- Male
- Female
- Other
- Prefer not to say

[]In this box, please leave any comments on the questionnaire you completed or on the use of hearing protection in general:

Please write your answer here:

Submit your survey.
Thank you for completing this survey.
